# Enhancing safety: Multi‐institutional FMEA and FTA on ‐based radio‐pharmaceutical therapy

**DOI:** 10.1002/acm2.14550

**Published:** 2024-11-13

**Authors:** Siju C. George, Santiago Aguirre, Nichole M. Maughan, Ranjini Tolakanahalli, E. James Jebaseelan Samuel, Sven L. Gallo, Jacqueline E. Zoberi, Yongsook C. Lee

**Affiliations:** ^1^ Department of Radiation Oncology Miami Cancer Institute, Baptist Health Miami Florida USA; ^2^ Department of Physics, School of Advanced Sciences Vellore Institute of Technology Vellore Tamil Nadu India; ^3^ Department of Radiation Oncology Herbert Wertheim College of Medicine, Florida International University Miami Florida USA; ^4^ Department of Radiation Oncology Intermountain Health Provo Utah USA; ^5^ Department of Nuclear Medicine University Hospitals Cleveland Medical Center Cleveland Ohio USA; ^6^ Department of Radiation Oncology Washington University School of Medicine St. Louis Missouri USA

**Keywords:** 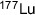
 radiopharmaceutical therapy, failure mode effects analysis, fault tree analysis, prospective analysis, process map, quality management

## Abstract

**Purpose:**

This study investigates potential failure modes and conducts failure mode and effects analysis (FMEA) and fault tree analysis (FTA) on the administration of 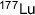 DOTATATE (LUTATHERA) and 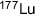 PSMA‐617 (PLUVICTO). The quality management (QM) process in radiopharmaceutical therapies (RPTs) requires collaboration between nuclear medicine (NM) and radiation oncology (RO) departments. As part of a multi‐institutional study, we surveyed various departments to identify and analyze failure modes, leading to a proposed comprehensive QM program. RPT teams in RO or NM clinics can benefit from this study by continually improving their practice.

**Methods:**

We reviewed the literature to investigate the administration of Pluvicto and Lutathera, focusing on prospective procedural failures and potential failure modes (PFMs) and their outcomes. We distributed an FMEA survey to multiple experienced centers in 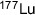
‐based RPTs and calculated risk priority number (RPN) for various PFM. We conducted an FTA using this information to pinpoint the root causes of potential failures.

**Results:**

The findings from the literature review and survey responses on the prospective study have identified several critical areas at risk of failure. These areas include non‐optimized treatment delivery, inadequate patient monitoring, and lack of safety training, leading to radiation contamination from the dose excreted by the patients after treatment administration. A segmented FTA was created based on the FMEA results, focusing on radiation contamination with a high RPN value.

**Conclusion:**

By identifying the root causes of failures and proposing targeted improvements to the existing QM measures, this analysis enhances safety in treatment delivery of 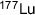
‐based RPTs. Given the limited number of prospective risk analysis studies in RPTs, our research addresses the necessity for more such studies and recommends methods to apply this study to other RPTs.

## 1 INTRODUCTION

Molecular radionuclide therapy is a treatment that uses imaging to evaluate its suitability and measure the response after treatment. It is referred to as a “theranostic” approach when the same molecule is used for imaging and therapy. Imaging maps target sites throughout the body using positron or gamma‐emitting radionuclides, while therapy delivers high radiation to the target lesions using beta particles, alpha particles, or Auger electrons. This targeted treatment approach for metastatic disease, which focuses on specific cancer cell targets, is expected to witness a rise in its clinical application. A growing understanding and research on cancer cells has led to the development of innovative radiopharmaceuticals for cancer treatment, with promising results. Radiopharmaceutical therapies (RPTs) are widely used in radiation medicine and have many applications in treating various diseases.^1^, ^2^ ^]^

In recent years, Lutetium‐177 (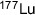) has gained popularity among researchers and clinicians as an unsealed radionuclide for some untreatable, metastatic cancers with poor prognoses. The positive results from the NETTER‐1 and VISION trials have re‐ignited the popularity of RPTs as a potential treatment option.^3^, ^4^ In 2018, LUTATHERA (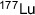
‐DOTATATE) was approved by the Food and Drug Administration (FDA) for targeted radiation treatment of gastroenteropancreatic neuroendocrine tumors in adults with positive hormone receptor somatostatin in the foregut, midgut, or hindgut. PLUVICTO (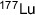 PSMA‐617) received FDA approval in May 2022 to treat patients with positive metastatic castration‐resistant prostate cancer undergoing androgen‐receptor pathway inhibition and taxane‐based chemotherapy. These radiopharmaceuticals contain the radioactive isotope 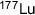 and selectively target somatostatin receptors and PSMA commonly expressed in neuroendocrine and prostate tumors. As of April 2024, LUTATHERA treatment is available at 284 healthcare systems in the United States (US), while PLUVICTO treatments are offered at 230 treatment centers.^5^

Despite their potential benefits, Lutathera and Pluvicto carry certain risks, including adverse reactions to the patients and inadvertent radiation risk to the public.^6^, ^7^ The scientific community must investigate all possible failure modes (FM) and their causes and effects for safe and efficient treatment delivery of these nascent RPTs. Identifying situations or reasons where treatments may not go as planned and the impact on the patient, staff, hospital, and the public cannot be overstated.^8^, ^9^, ^10^ These innovative therapies must be implemented with a robust quality management (QM) program to ensure patient safety and effectiveness.

“Report No. 283 ‐ The report of Task Group 100 (TG‐100) of the American Association of Physicists in Medicine (AAPM): Application of risk analysis methods to radiation therapy quality management (2016)” proposes risk analysis for QM in radiation therapy. The report recommends systematically evaluating potential failure modes (PFMs) throughout radiation therapy procedures to better allocate QM resources for optimal patient safety and care quality. The report suggests using risk‐based analysis techniques, such as failure mode and effects analysis (FMEA) and fault tree analysis (FTA), to assess different processes of radiation therapy procedures and enhance the safety and efficiency of the treatment.^11^ Hermann et al. recommend established risk analysis methods, such as FMEA or FTA, to set up RPT programs.^9^ To ensure the safety of RPT, conducting a thorough risk assessment is imperative, and FMEA/FTA can achieve this. Even though FMEA has been used extensively for risk assessment in radiation medicine, only a few studies have applied it to RPT.^12^, ^13^, ^14^, ^15^ FMEA and FTA needs to be utilized more in RPT, and more studies employing this approach are warranted. The TG‐100 report emphasizes collaboration among radiation treatment team members to ensure adequate QM. It offers guidance for implementing QM programs and outlines a structured methodology for analyzing clinical processes and creating site‐specific QM programs. Prospective risk analysis aims to identify potential risky steps before failures occur, followed by designing new processes or modifying existing ones to reduce potential failures. Medical safety and quality can be improved through risk‐based approaches for assessing and preventing failures.^11^

A literature review was conducted to analyze the administration of Pluvicto and Lutathera and identify potential procedural failures and their consequences.^8^, ^16^, ^17^, ^18^, ^19^ After reviewing the intricacies of setting up the Lutathera treatment program in an outpatient setting, Maughan et al. conducted a workflow risk assessment using FMEA to identify PFMs that could lead to incorrect dosage or radiation contamination.^14^ The manuscript was prepared before the FDA approved Pluvicto treatments, so additional PFMs related to Pluvicto treatments must be identified for a comprehensive 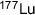 RPT risk analysis. Zoberi et al. described the complex details and steps to implement a Pluvicto program in a healthcare facility.^20^ The work highlighted different infusion methods and the potential challenges of setting up the program. The FMs encountered through the literature review could be generalized to any healthcare facility, highlighting the need for an extensive FMEA that could be useful across multiple institutions. A recent publication analyzed the workflow and steps for 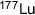
‐based RPT and presented checklists for establishing and optimizing Lutathera and Pluvicto RPT programs, ensuring quality improvement in every step.^21^ The manuscript recognized the limitations of a checklist‐only approach and advocated using more comprehensive risk analysis to identify and preempt potential failures. The study emphasized the importance of using FMEA methodology to identify and prevent any PFM in 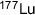
‐based RPT following AAPM's TG‐100 recommendations.

The treatment protocols and practices are mostly similar across 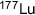 RPT treatment centers in the US. The supplier of these RPTs (Novartis, Basel, Switzerland) provides training for implementing the program, and a prescribed methodology must be followed for the administration of Lutathera and Pluvicto in any treatment centers, as they comply with the relevant state licenses. This ensures minimal variation between departments delivering the currently approved 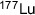
‐based RPTs.^8^, ^14^ An integrated approach between nuclear medicine (NM) and radiation oncology (RO) departments is necessary to improve the quality of RPT programs. Quality improvement projects in a radiopharmaceutical treatment center (NM or RO) should focus on accurate and efficient patient care, patient/staff safety, and patient experience. A quality control process ensures that equipment functions correctly. In contrast, quality improvement is used to solve problems in a structured and data‐driven manner to provide better long‐term solutions.^22^ The joint efforts will develop a sustainable QM program and foster collaboration among RPT treatment centers, even if minor differences in program structures pose challenges. Our study presents the results of a multi‐institutional (and multi‐departmental) FMEA and FTA for risk analysis on the current FDA‐approved 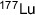
‐based RPTs, along with recommendations for enhancing the QM program.

## 2 METHODS

### 2.1 Process mapping

**FIGURE 1:**
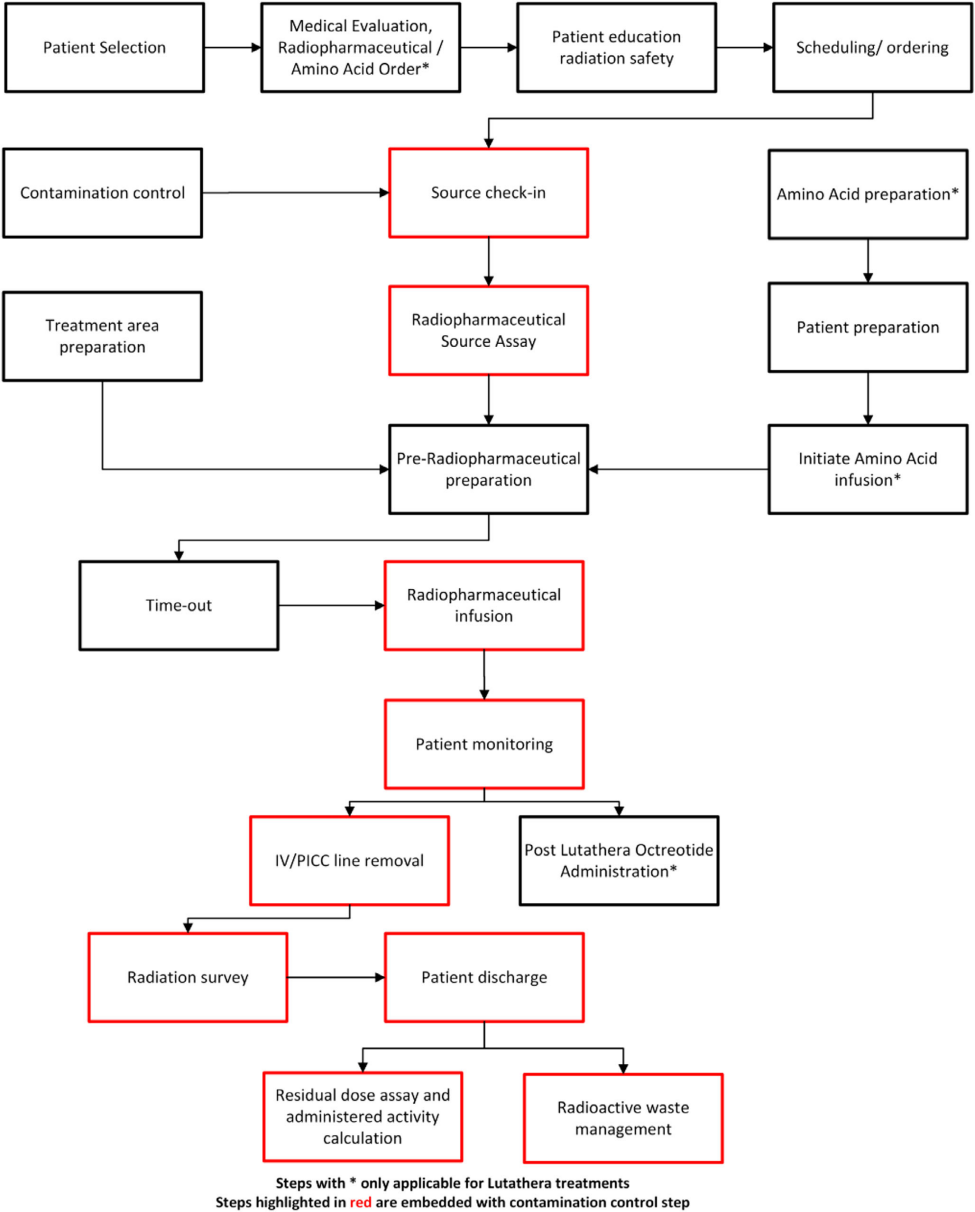
Lutathera and Pluvicto treatment process mapping.

We adopted a systematic approach, placing all components involved in administering Lutathera and Pluvicto in a process map as seen in Figure 1. The entire Lutathera and Pluvicto treatment process was divided into 21 modules demonstrating the treatment dynamics at various departments where 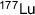
‐based RPTs are commonly administered. Of the 21 listed items, three are relevant (amino acid‐related items) only for Lutathera treatments, while the remaining 18 apply to both Lutathera and Pluvicto treatments.

The professionals responsible for each step were determined based on their relevant roles within the healthcare system where the study was initiated. The identified roles were:

1. Authorized User (AU): Select appropriate patients, prescribe and deliver treatments, and follow up with side effects management if detected.
2. Radiation Safety Officer (RSO): Supervises radiation safety responsibilities and ensures compliance with state license requirements.
3. Medical Physicist: Responsible for all aspects of treatment delivery and patient and staff safety; oversees medical records and safety documents.
4. Medical Physics Assistant (MPA): Assist medical physicists with dose assay, patient and room surveys, treatment room preparation, post‐treatment cleaning, radioactive waste storage, and so forth.
5. Registered Nurse: Patient care from arrival to the department until discharge, monitor patient safety‐related items, identify acute side effects, assist treatment delivery and documentation with licensed staff.
6. Radio‐pharmacist: Amino Acid preparations and pharmaceutical consultations.
7. Administrative staff: Scheduling patients and ordering doses for appropriate treatments; they support general practice management.
8. Treatment delivery technologist (NM or RO): RPT administration with AU and Medical Physics support. Support Physics and MPA for treatment preparation and post‐treatment responsibilities.

**FIGURE 2:**
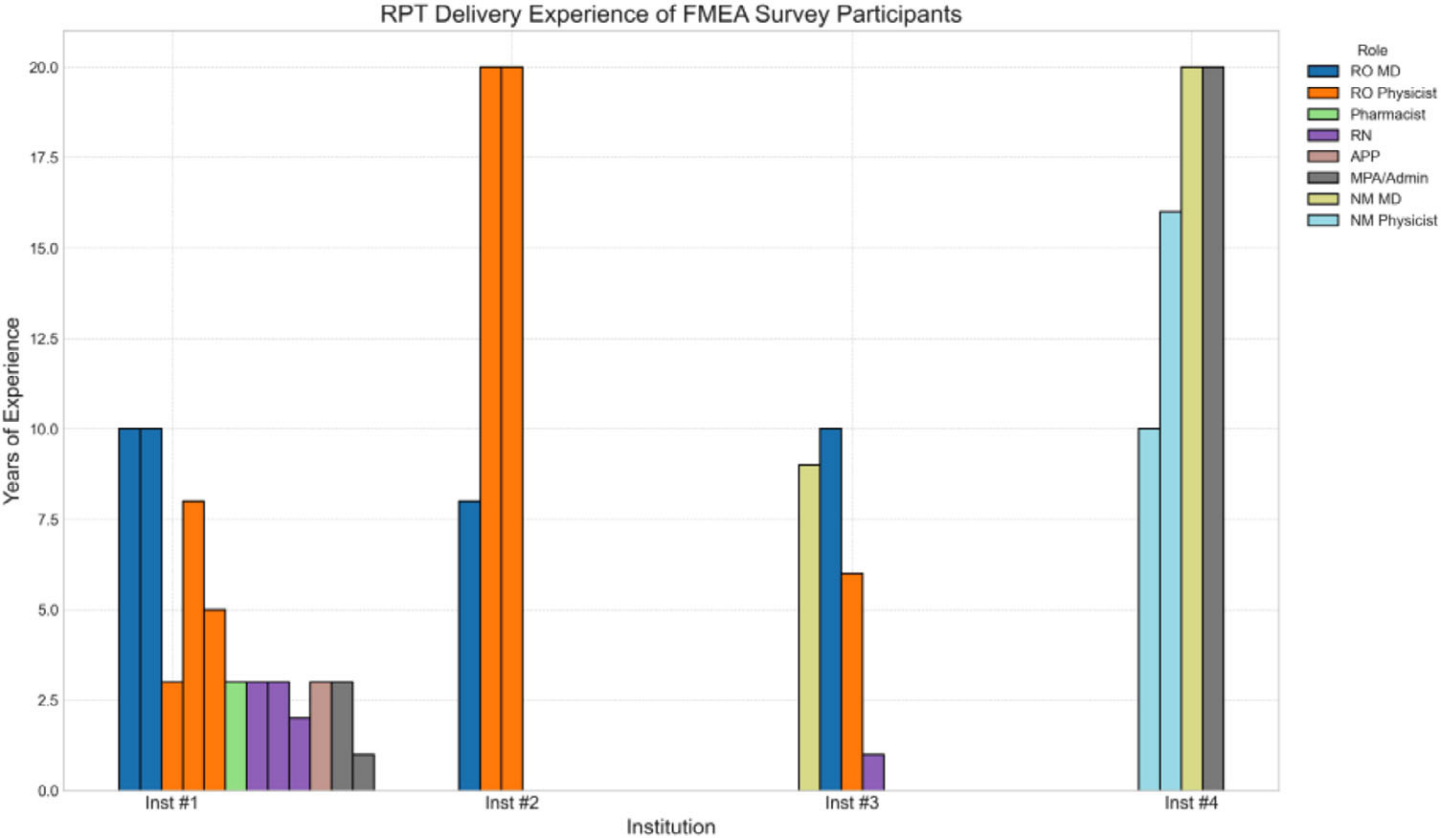
Experience in RPT delivery of 23 survey participants. To reduce the size of the graph, more than 20 years of experience were set as 20.

The research team contacted other high‐volume treatment centers that administer 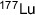
‐based radiopharmaceuticals to collaborate on this multi‐institutional study. They recruited additional collaborators listed in the Acknowledgment section. The survey included two RO departments (Institution#1 and Institution#2), one treatment center (Institution#3) with a hybrid model supporting both NM and RO departments, and one NM department (Institution#4) delivering 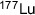
‐based RPT. The years of clinical RPT experience for the 23 survey participants are detailed in Figure 2. Additionally, over the past 5 years, Institution#1 administered approximately 200 infusions, Institution#2 completed 900 infusions, Institution#3 delivered 400 infusions, and Institution#4 provided 800 infusions of 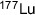
‐based RPT. The collected data is anonymized to alleviate any concerns the survey team members may have about sharing their scholarly opinions on potential risk analysis scores. This should help them feel more comfortable sharing quality management (QM) limitations at their treatment centers and motivate them to focus on continuous improvements.

### 2.2 FMEA

#### 2.2.1 Identifying FM, causes, effects, and setting up the survey

As a part of this prospective study, a survey was conducted to enlist 117 unique PFMs related to Lutathera and Pluvicto, covering 22 distinct steps (details are available in the supplementary materials).^23^ It was determined that these steps were necessary by investigating and expanding the process map listed in Section 2.1. The survey lists the relevant PFMs, connects them to the causes and potential effects of failure, and aims to score their Occurrence (O), Severity (S), and Detectability (D) values.

- O (occurrence) describes the likelihood that a particular cause for the specified failure mode exists. A higher score indicates a higher likelihood of occurrence.
- S (severity) describes the severity of the effect of failure on the final process outcome resulting from the failure mode if it is not detected or corrected. The higher the score, the greater the severity.
- D (lack of detectability) describes the likelihood that the failure will not be detected to prevent an event (effect of failure). Higher scores indicate lower detectability or more difficulty in detecting the error.

Since PFMs are associated with various effects and causes of failure, the FMEA scoring sheet had 172 lines encompassing all professional activities used in Pluvicto and Lutathera RPT. Based on their potential effects of failure, the data was categorized into two groups for analysis: (1) radiation‐related (radiation contamination, public safety, suboptimal results, side effects, etc.) and (2) administration‐related (regulatory non‐compliance or wasted resources). Some PFMs may result in effects that fall into both categories. For example, the failure mode, “Room release survey, not performed,” can result in radiation contamination and regulatory non‐compliance categories. Causes of failures falling in the same category, such as human errors, inadequate training, or lack of supervision, were classified under the term “personnel performance lapse” to simplify the FMEA sheet and expedite scoring. The side effects were assessed based on the severity of the reported adverse events in the respective trials. These are discussed in the pharmaceutical information leaflet in each dose package delivery to the treatment center.^6^, ^7^ The side effects were directly attributed to the RPTs under consideration, without bias to other variables such as patient age or comorbidity.

We used a qualitative scale inspired by ‘Table II in TG‐100,’ which uses a scale of 1–5.^11^ Table 1 was developed following the training provided by the AAPM “TG‐100 Webinar Series: Risk‐Informed Quality Management” in August/September 2022. This also made the scoring process more straightforward for the FMEA team members. To ensure consistency and accuracy in scoring, we trained all participants on interpreting O, S, and D values with a detailed explanation of how to score the S Value based on the attached reference Table 2.

**TABLE 1:** TG‐100 table II modified, qualitative attributes and ranks.

	Occurrence	Severity		Detectability
Rank		Radiation‐related	Administration‐related	
1	Cause unlikely	Negligible or no impact on patient	Minor administrative inconvenience or resource wastage	Obvious
2	Relatively few failures	Minor side effects, temporary discomfort	Item needs attention, but does not breach regulatory compliance	Easy to detect
3	Occasional failures	Significant side effects, manageable long‐term effects	Major administrative impact (e.g., dose spillage, needs emergency management)	Detectable with careful review
4	Repeated failures	Severe side effects requiring intervention (but not life‐threatening)	Severe administrative consequences (e.g., severe resource wastage)	Detectable with thorough investigation
5	Failures highly likely	Life‐threatening or fatal side effects	Catastrophic administrative consequences (e.g., legal actions, loss of license)	Impossible to detect

**TABLE 2:** Effect of failure terms and severity descriptions with scoring guideline.

**Effect of failure**	**S Value**	**Description**
Public safety	2–3	Public Safety includes radiation contamination to children and vulnerable populations
Suboptimal results	2–4	Deviation from Rx activity more than 10%
Side effects	1	Patient discomfort
	2	Nausea, vomiting, diarrhea, myelosuppression, flushing, extravasation.
	3	Tissue necrosis, bronchospasm, hypotension, kidney injury, liver injury, infertility.
	4	Secondary cancer (leukemia, Myelodysplastic Syndrome).
	5	Death
Radiation contamination	2–4	Exposure to unwanted radiation or radionuclides.
Wasted resources	1–3	Inconvenience, delay in treatment, staff staying late, waste of medical supplies, or cancellation and wasting the drug.
Regulatory non‐compliance	2–4	Going beyond allowed practices according to the agreed terms in the license, If the delivered dose differs from the prescribed dose by ±20% or more OR wrong route/medicine/patient (reportable medical event).

The multi‐institutional survey received a significant response from a diverse data‐scoring team. Active participation by 23 individuals from varying professional backgrounds significantly enhanced the FMEA process, enabling a thorough analysis of the PFMs in the 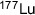
‐based RPT. In addition to one pharmacist, six AU physicians practicing RO or NM specialties, three registered nurses, two medical physics assistants, eight medical physicists (RO or NM department, one of them is RSO), two advanced practice providers, and one NM technology administrator participated in the survey. This composition provided a wide‐ranging perspective on 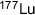
‐based treatments in multi‐disciplinary treatment centers. Additionally, the initiative benefited from participants' expertise from four leading healthcare institutions in the US, who contributed their specialized clinical and technical knowledge to this study as indicated by Figure 2.

#### 2.2.2 Evaluating survey results and RPN calculations

**FIGURE 3:**
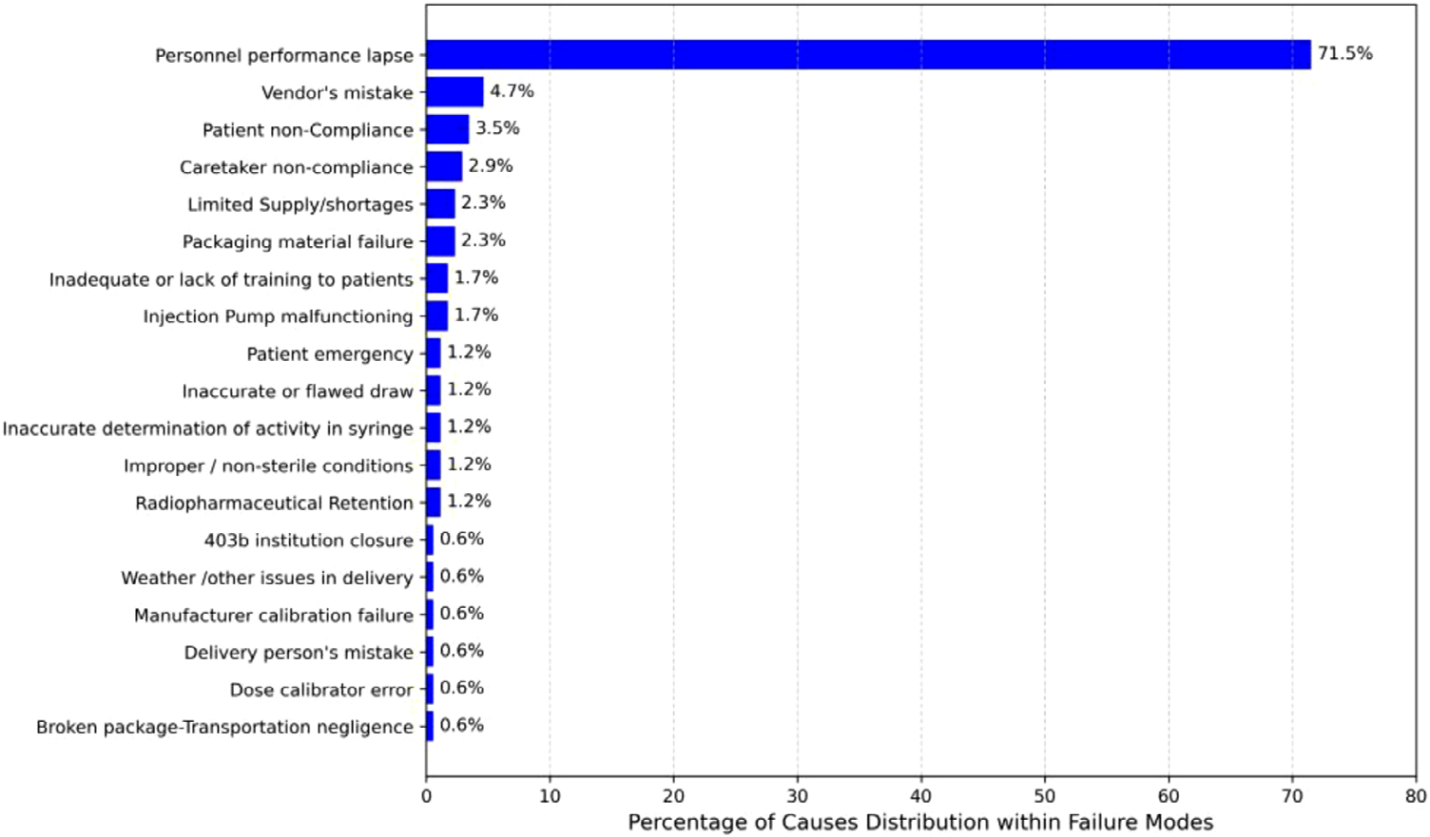
Total FME distribution based on 19 different causes.

**FIGURE 4:**
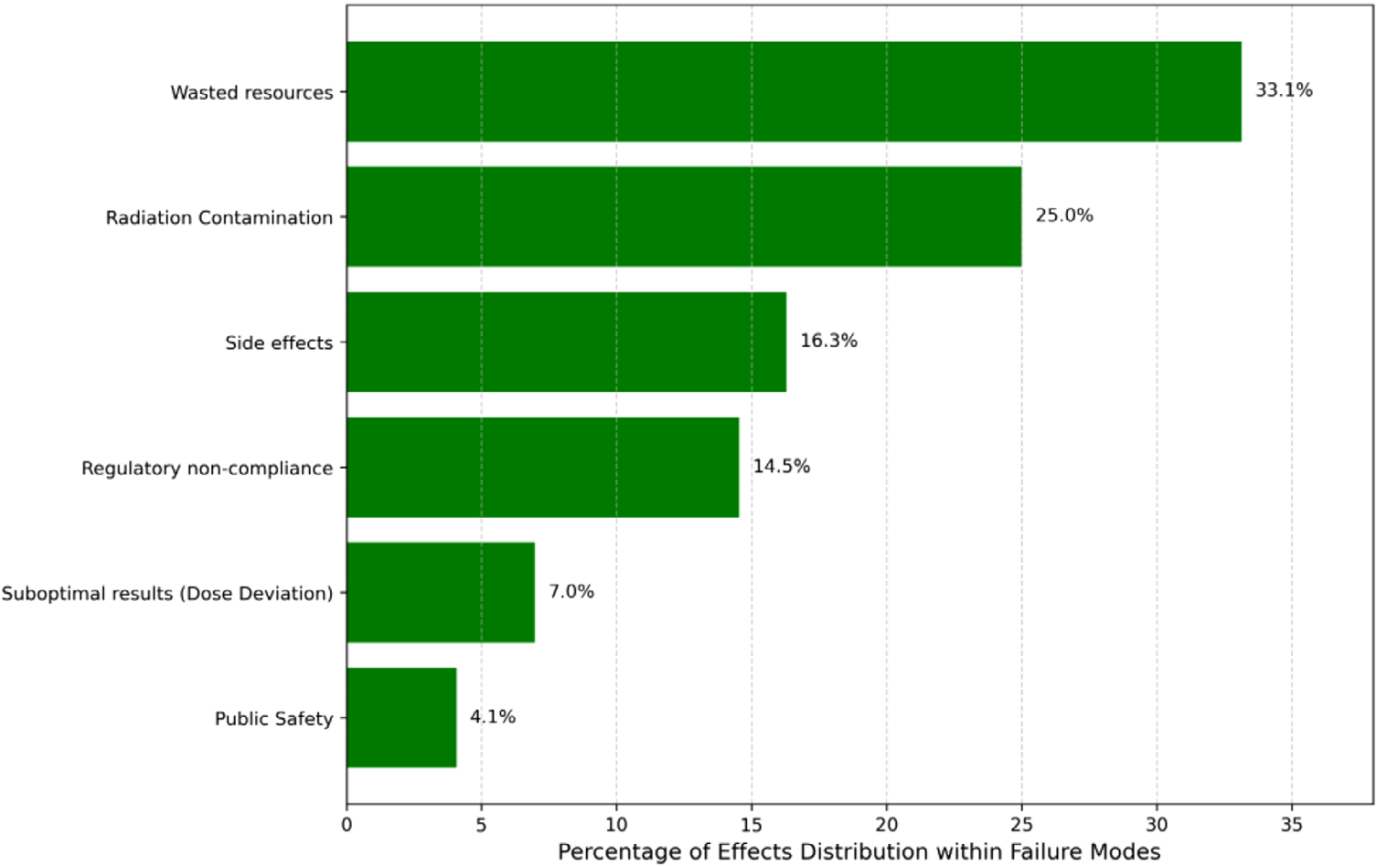
Total FME distribution with six different effects.

A structured workflow was established to collect O, S, and D scores on FMs from the survey of all participating institutions. Each participating institution involved at least three professionals from their treatment centers in the data collection. Participants were allowed to score items related to their professional expertise and areas where they could apply educated judgment since all the 117 PFMs were irrelevant to every participant. The survey participants addressed 156 out of the 172 line items. The authors collaboratively scored the remaining 16 unattended survey items during data evaluation. The average O, S, and D values from each institution's participants on every PFM were extracted into a new data analysis sheet using Python scripts. Sorting and color‐coding tools were applied to the data analysis sheet to categorize the FMs into administration or radiation‐related segments. The “Cause of failure” column cataloged all potential causes attributed to each failure mode. Figures 3 and 4 list the six effects and 19 causes of failures concerning the total analyzed 172 items.

Finally, the risk priority number (RPN) for 172 surveyed items was calculated independently for each institution as the product of the averaged O, S, and D values using Equation (1). According to the scoring scale used in this study, RPN values range from 1 to 125.

(1) $${\mathrm{RPN}}_{\text{ave}}=O_{\text{ave}}\times S_{\text{ave}}\times D_{\text{ave}}$$

### 2.3 FTA

We prioritized PFMs based on calculated RPNs and illustrated an FTA using Microsoft Vizio. According to the FMEA survey results, we focused on one effect, “Radiation Contamination,” with a high RPN value. Identifying PFMs for corresponding operational steps allowed us to trace these effects to their root causes. Using data from the FMEA survey and expert insights, we analyzed how and why each step failed. The RPN numbers were used with qualitative analysis to evaluate or propose targeted risk mitigation strategies for the treatment delivery process. Mitigation strategies or QM improvement recommendations for each connected item were identified by marking QM activities on the FTA. Existing QM strategies could address some of the FMs discussed in the study. If there are gaps, we suggested recommendations for further inquiry or mitigation to prevent identified failures and proactively resolve errors in the RPT clinics.

## 3 RESULTS

### 3.1 FMEA

Figures 3 and 4 provide a comprehensive visual representation of the distribution of FM categorized by their underlying causes and associated effects, offering an overview of the identified risks within the 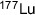
‐based RPT treatment paradigm. A well‐established RPT program may not experience these FMs as often as a newly developed one. Out of the 172 surveyed items, a noteworthy proportion, comprising 28 PFMs (16.28%), was attributed to side effects experienced by patients. The identified side effects of Lutathera and Pluvicto included a spectrum of adverse reactions that ranged from mild discomforts, such as nausea, vomiting, fatigue, and Xerostomia, to more severe manifestations, including hepatotoxicity, hematologic and renal toxicities.^2^, ^6^, ^7^, ^24^ Another significant concern identified from the analysis was regulatory non‐compliance, which accounted for 25 PFMs (14.53%). Regulatory non‐compliance instances encompassed deviations from established regulations, including dosage errors beyond permissible limits, incorrect administration routes, or treatments that may lead to reportable medical events. The analysis also identified 57 PFMs (33.14%) linked to the inefficient utilization of resources. These PFMs led to inconveniences, treatment delays, wasted medical supplies, and drug cancelations, underscoring the importance of resource optimization in treatment delivery.

The present study disclose that 12 (6.98%) PFMs were associated with sub‐optimal treatment outcomes or dose deviations exceeding 10% of the prescribed dose, thereby emphasizing the criticality of precise dosing in achieving therapeutic efficacy. Notably, 43 PFMs (25%) were attributed to the effects of radiation contamination emanating from errors during handling of the RPT or patient/caregiver failures to safely handle the contaminated excretions from the patient after treatments that may result in inadvertently spreading radiation contamination, ultimately exposing patient, caregivers or the public to unnecessary radiation doses. The study also identified an additional 7 PFMs (4.07%) directly related to public safety, potentially exceeding ALARA (as low as reasonably achievable) levels.

**FIGURE 5:**
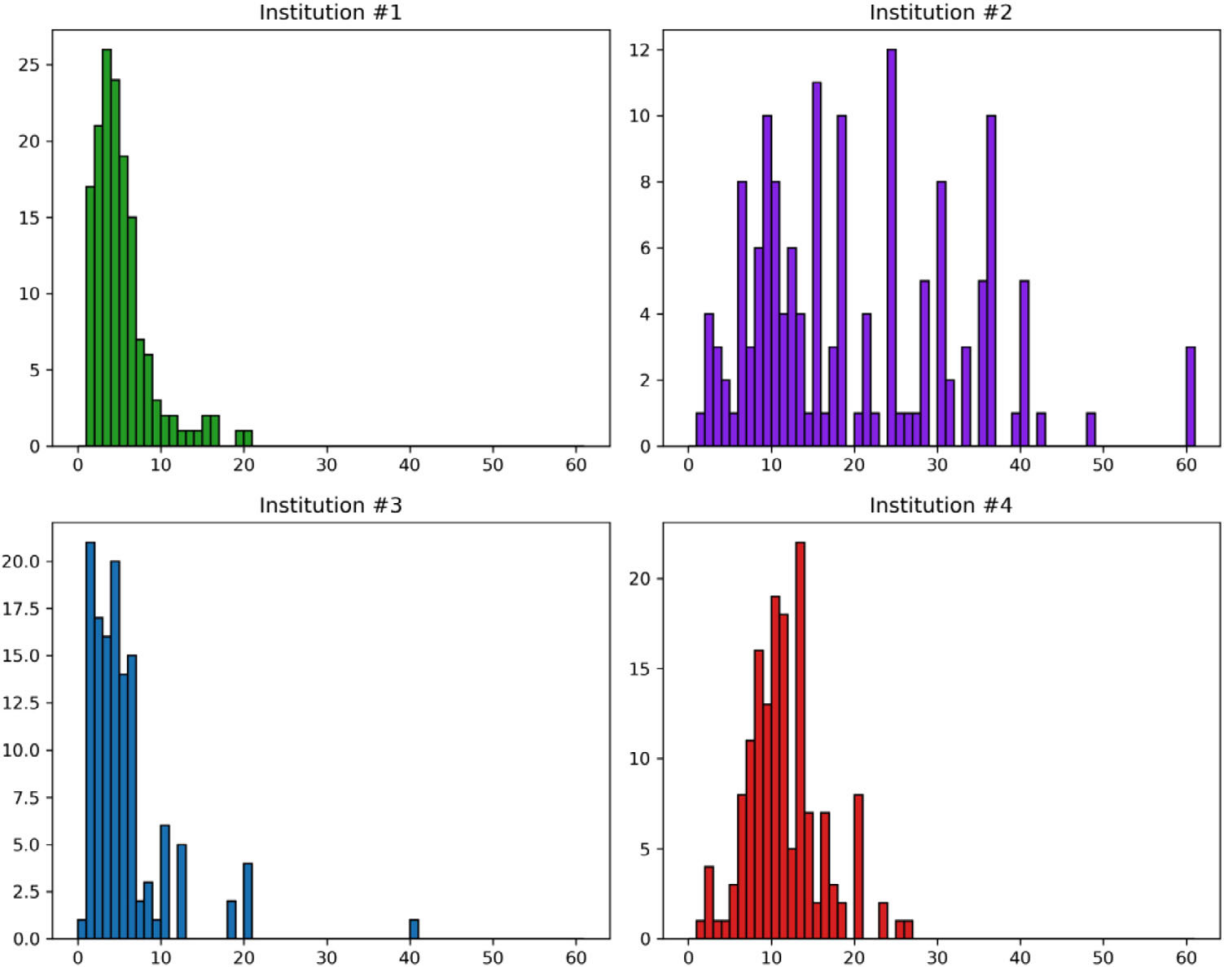
Distribution of RPN scores among different participating institutions delivering 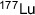
‐based RPT: Institution#1 and Institution#2 are RO departments. Institution#3 with a hybrid model supports both NM and RO departments. Institution#4 is an NM department.

Figure 5 depicts the distribution of RPN scores among various participating institutions and separates the differences in practice between different departments. The combined comparison or evaluation of RPN values from separate institutions does not add value to this study because QM methodologies and practice guidelines may vary. Note that the RPN score distributions from Institution #2 had a wide range (1 to 60) while Institution #1 scored low numbers (1 to 21), similar to Institution #4 (1 to 26). Details of the O, S, and D scoring, RPN numbers, and data analysis are available in the supplementary material.^23^

Table 3 presents details of the highest five RPN values on radiation‐related effects. Participating institutions identified radiation contamination and public safety as significant concerns in the radiation‐related effects category. For instance, the PFMs “The patient travels using public transportation” and “caregivers not following adequate safety precautions” scored high RPNs from multiple institutions. Similarly, Table 4 shows the top five highest RPN values with an administrative impact. In the administration‐related category, two centers identified “Lack of availability of pre‐mixed amino acids” as a significant issue leading to wasted resources. “Personal performance lapses” leading to regulatory non‐compliance also scored high RPN values from all the participating institutions.

**TABLE 3:** Top 5 FM per institution for radiation‐related effects, yellow rows indicate FM presenting in the top 5 RPN items for more than one institution.

Failure mode	Cause	Effect	$O_{avg}$	$S_{avg}$	$D_{avg}$	${\mathrm{RPN}}_{avg}$
Institution #1						
The patient travels using public transportation	Patient non‐compliance	Public safety	1.67	2.67	4.67	20.74
Patient did not follow instructions	Patient non‐compliance	Public safety	1.83	2.67	4.00	19.56
Patient personal hygiene inadequate (less than daily showering)	Caretaker non‐compliance	Radiation contamination	1.50	2.50	4.50	16.88
Instructions for patients with limited mobility not followed by caretaker	Caretaker non‐compliance	Public safety	1.50	2.50	4.17	15.63
Assisted living patients' caregivers not following adequate safety precautions	Caretaker non‐compliance	Public safety	1.33	2.67	4.00	14.20
Institution #2						
The patient travels using public transportation	Patient non‐compliance	Radiation contamination	4.00	3.00	4.00	48.00
Inaccurate measurement	Inaccurate or flawed draw	Radiation contamination	2.50	4.00	4.00	40.00
Pump failed to deliver full dose	Personnel performance lapse	Suboptimal results	2.00	5.00	4.00	40.00
Incontinent patients' urine spills‐ not adequately prepared	Personnel performance lapse	Radiation contamination	3.00	4.00	3.00	36.00
Assisted living patients' caregivers not following adequate safety precautions	Caretaker non‐compliance	Radiation contamination	4.00	3.00	3.00	36.00
Institution #3						
Assisted living patients' caregivers not following adequate safety precautions	Caretaker non‐compliance	Radiation contamination	2.00	4.00	5.00	40.00
The patient travels using public transportation	Patient non‐compliance	Radiation contamination	1.00	4.00	5.00	20.00
Incontinent patients' urine spills‐ not adequately prepared	Personnel performance lapse	Radiation contamination	1.00	4.00	5.00	20.00
Staff not surveyed prior to leaving room	Personnel performance lapse	Radiation contamination	2.00	3.00	3.00	18.00
Cart not surveyed for contamination	Personnel performance lapse	Radiation contamination	2.00	2.00	3.00	12.00
Institution #4						
Did not deliver complete activity (more than acceptable activity still in lines and vials)	Personnel performance lapse	Suboptimal results	2.50	3.50	3.00	26.25
Not monitoring for IV line occlusion	Personnel performance lapse	Radiation contamination	2.33	3.00	3.67	25.67
Level of fluid in vial not checked during infusion	Personnel performance lapse	Radiation contamination	2.33	3.00	3.33	23.33
No monitoring or incorrect monitoring of exposure rate of vial vs. patient	Personnel performance lapse	Radiation contamination	2.33	3.33	3.00	23.33
No monitoring for extravasation, discomfort of patient	Personnel performance lapse	Side effects	2.33	3.00	3.00	20.97

**TABLE 4:** Top 5 FM per institution for administration‐related effects, yellow rows indicate FM presenting in the top 5 RPN items for more than one institution

Failure mode	Cause	Effect	$O_{avg}$	$S_{avg}$	$D_{avg}$	${\mathrm{RPN}}_{avg}$
Institution #1						
Lack of availability of pre‐mixed amino acids	403b institution closure	Wasted resources	2.00	2.25	2.00	9.00
Wrong dose/vial	Personnel performance lapse	Regulatory non‐compliance	1.17	4.17	1.67	8.10
Wrong radionuclide	Personnel performance lapse	Regulatory non‐compliance	1.00	4.33	1.83	7.94
Staff not surveyed prior to leaving room	Personnel performance lapse	Regulatory non‐compliance	1.25	2.50	2.50	7.81
Patient did not receive clear release instructions	Personnel performance lapse	Regulatory non‐compliance	1.25	2.50	2.25	7.03
Institution #2						
Lack of availability of pre‐mixed amino acids	403b institution closure	Wasted resources	3.00	5.00	4.00	60.00
Amino Acid order not placed by provider	Personnel performance lapse	Wasted resources	3.00	5.00	4.00	60.00
Delivered to wrong physical location	Delivery person's mistake	Wasted resources	3.00	4.00	3.50	42.00
Inaccurate measurement	Inaccurate determination of activity in syringe	Wasted resources	2.50	4.00	4.00	40.00
Patient was released without Survey	Personnel performance lapse	Regulatory non‐compliance	2.50	4.50	3.50	39.38
Institution #3						
Staff not surveyed prior to leaving room	Personnel performance lapse	Regulatory non‐compliance	2.00	3.00	3.00	18.00
Pump failed to deliver full dose	Injection Pump malfunctioning	Regulatory non‐compliance	1.00	4.00	3.00	12.00
Patient NOT scheduled for lab works and visit one week before the treatment	Personnel performance lapse	Wasted resources	2.00	3.50	1.00	7.00
Contaminated items placed in regular waste	Personnel performance lapse	Regulatory non‐compliance	2.00	3.00	1.00	6.00
Inaccurate measurement of residual activities	Personnel performance lapse	Regulatory non‐compliance	1.00	2.00	3.00	6.00
Institution #4						
Batch not released by the manufacturer	Vendor's mistake	Wasted resources	2.00	3.00	3.00	18.00
Wrong dose/vial	Personnel performance lapse	Regulatory non‐compliance	1.33	4.00	3.33	17.78
Wrong patient	Personnel performance lapse	Regulatory non‐compliance	1.33	4.00	3.33	17.78
Lines (IV or PICC) not primed and checked for flow before Lu administration	Personnel performance lapse	Wasted resources	1.67	3.33	3.00	16.67
Wrong radionuclide	Personnel performance lapse	Regulatory non‐compliance	1.33	3.67	3.33	16.30

### 3.2 FTA

The highest‐scoring RPNs pointed to “radiation contamination” as a primary effect of concern for most institutions in the study. Therefore, we focused solely on this effect in our FTA to avoid redundant elaboration and discussion. After identifying the specific undesired effect, we singled out the critical step #16 (the data analysis tab in the supplementary material enlists the 22 distinct steps), “contamination control,” which played a decisive role in such events. The next step was to examine all the possible PFMs related to contamination control that could lead to radiation contamination. For each failure mode, all potential causes were explored to understand the factors that could contribute to this particular effect.^23^

**FIGURE 6:**
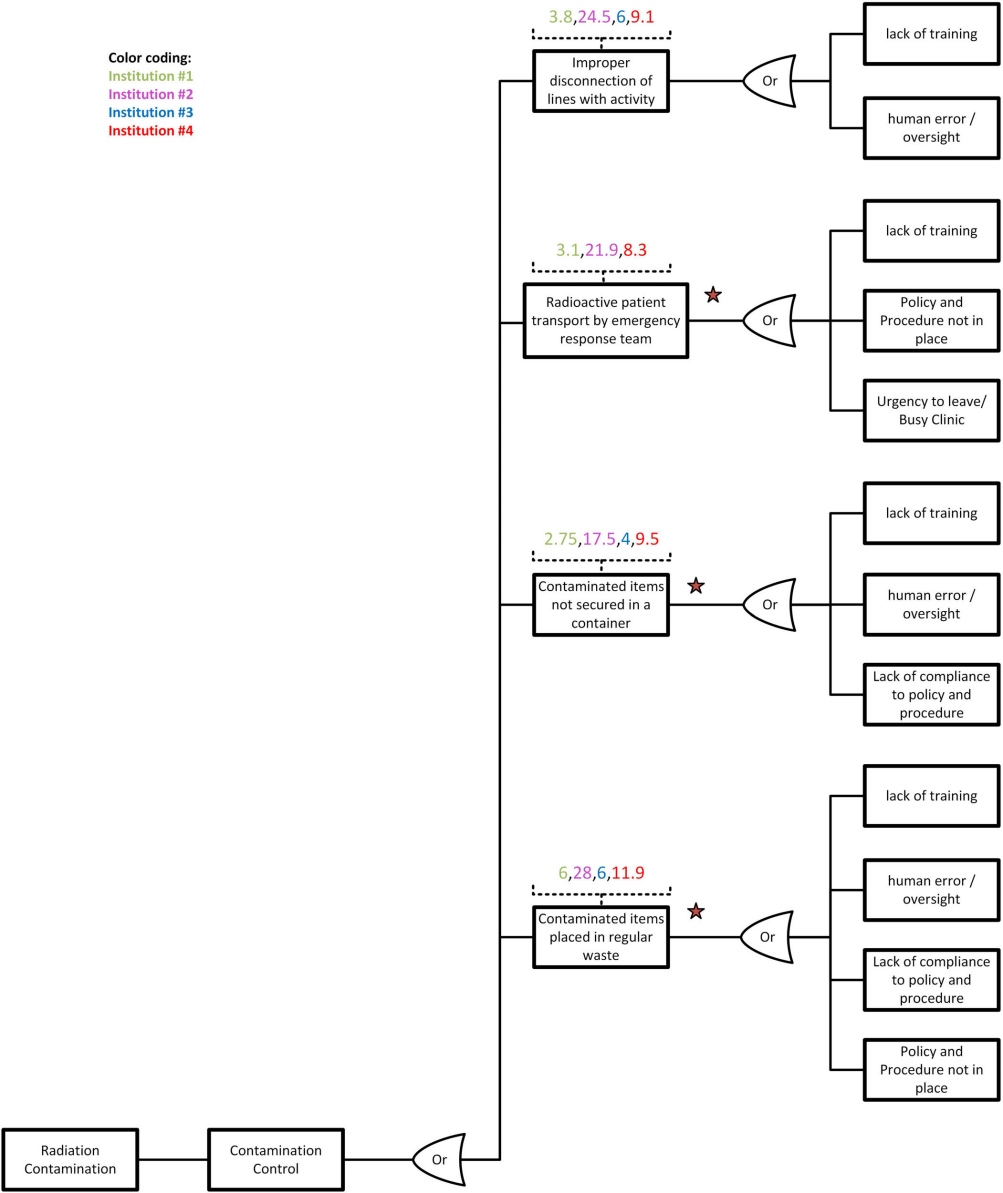
Fault tree analysis (top section) of radiation contamination focused on contamination control step (#16). The numbers above each failure mode represent the average RPN reported by different institutions. The stars indicate existing QM strategies applied at participating institutions.

**FIGURE 7:**
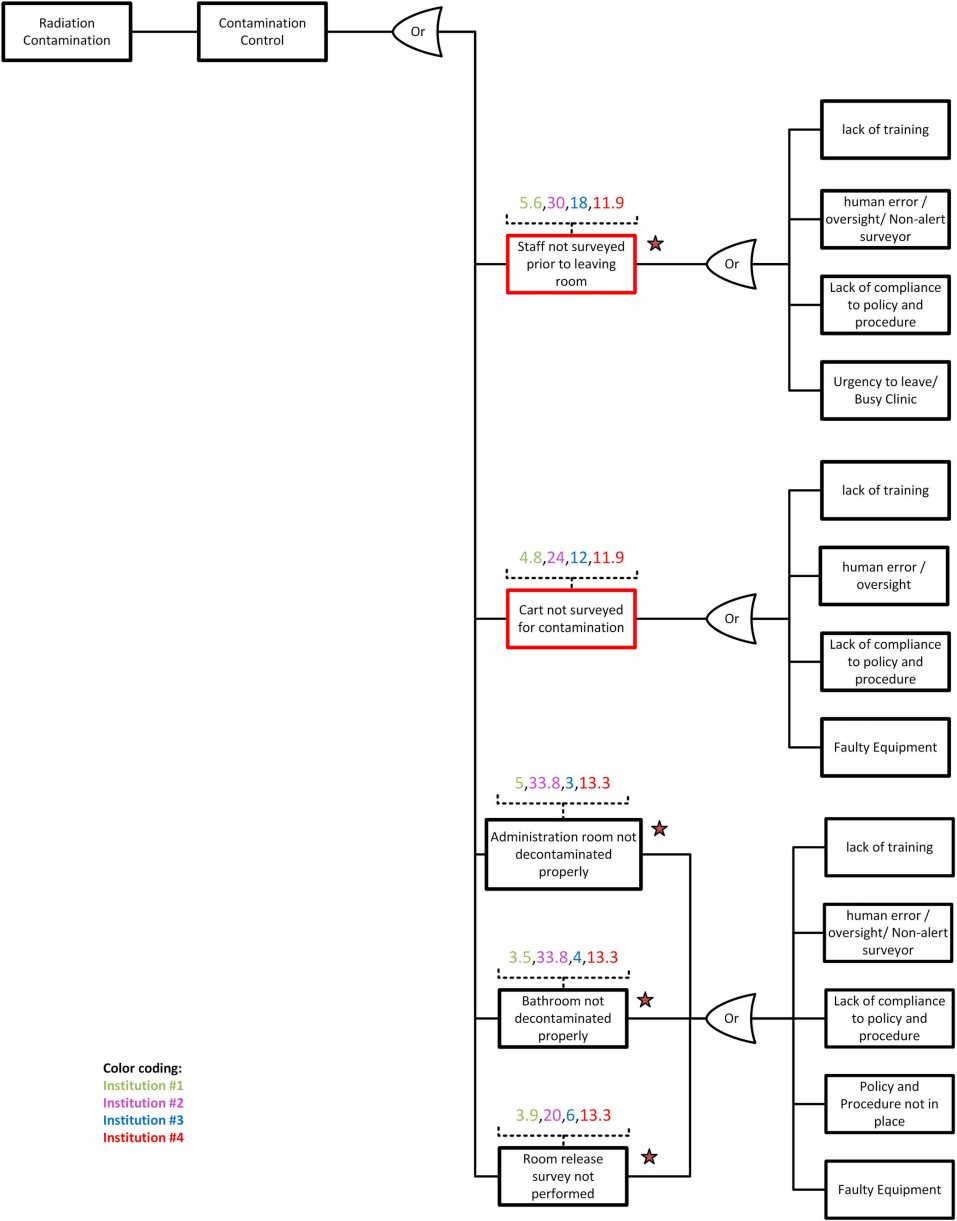
Fault tree analysis (bottom section) of radiation contamination focused on contamination control step (#16). The numbers above each failure mode represent the average RPN reported by different institutions. The stars indicate existing QM strategies applied at participating institutions. RPNs among the top 10% from any institution are indicated by colored boxes.

After identifying the FM and related causes of failures, the FTA focused on identifying critical paths and vulnerabilities in the contamination control step. This phase highlights the most significant risk factors and pinpoints where interventions could reduce the likelihood of radiation contamination. Additionally, integrating current QM measures into the FTA provided a snapshot of the existing safeguards against contamination. This integration served a dual purpose: (1)Validating the effectiveness of current practices and (2) Identifying potential gaps where additional QM protocols could be introduced. Nearly 70% of the PFM displayed in the developed FTA already have existing QM systems, marked with a star on the FTA Figures 6 and 7.

Robust QM is essential to ensure safe and effective 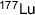 RPTs by upholding treatment standards and reducing potential risks. Although existing protocols provide a foundation for safety, periodic evaluation and reinforcement are required to address gaps. Attention to detail in every step, from surveying and accepting the source, preparing the room, treating the patient, discharging them, and safely storing radioactive waste, is warranted for safely practicing RPT.^19^ These processes are supplemented by individualized checklists tailored to Lutathera and Pluvicto treatments' specific requirements, exemplifying our unwavering commitment to ensuring the highest standards of safety and efficacy in 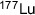
‐based RPT delivery.^21^ A comprehensive list of mitigation strategies for the FMs discussed in this study is presented in Table 5 based on the extensive experience of participating institutions over the past 5 years and following recommendations from different publications.^8^, ^9^, ^14^, ^20^, ^22^ The strategies presented here will be helpful tools for new and experienced users intending to improve their QM program for 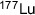
‐based RPT.

**TABLE 5:** Quality management items relevant to multiple FMs and the step(s) for ‐based RPT.

**Failure mode**	**Mitigation strategy**	**Step(s) addressed**
Radioactive contamination on packaging	Survey/leak test when source arrives	Source check‐in
Inaccurate measurement	Source assay conducted by a two‐person team	Source assay
Inadequate decay‐in‐storage and waste disposal program.	Source acceptance/radioactive waste log periodic evaluations	Source check‐in & radioactive waste management
Inadequate decay‐in‐storage and waste disposal program.	Radioactive waste materials management checklist and hot lab etiquette	Radioactive waste management
Missed area prep covering with chucks & paper	Room preparation and verification before the patient arrives	Area preparation
Public safety	Source transport in a proper container through the back corridor	Contamination control
Public safety	Time out before RPT infusion	Special category
Lines (IV or PICC) not primed and checked for flow before Lu administration	Needle insertion verification by a second person	Patient preparation
No monitoring or incorrect monitoring of exposure rate of vial vs. patient	Blue‐tooth connected Survey meter display	Radiopharm delivery
Incorrect documentation of exposure rate	Patient release survey documentation	Patient discharge
Most of the FMs	Separate and comprehensive treatment day checklists for Lutathera and Pluvicto,^21^	All steps
Bathroom not decontaminated properly	Create a dedicated bathroom for the use of Lu‐177‐based treatments	Contamination control
Radioactive patient transport by emergency response team	Emergency team development for RPT with adequate training for first responders,^10^	Contamination control
Administration room not decontaminated properly & Room release survey	Create checklists for releasing the treatment area for others to use	Contamination control
The patient travels using public transportation	Using new ways for patient education/comprehension of rad safety—visual coaching or videos in different languages	Special category
The patient travels using public transportation	Reinforcement of transportation arrangements with the patient/caretaker before treatment delivery	Special category
Assisted living patients' caregivers not following adequate safety precautions	Evaluation of understanding of the Rad Safety principles of the patient or caretaker and Post administration transportation issues	Special category
Assisted living patients' caregivers not following adequate safety precautions	Questionnaires and materials for the living arrangement with clear instructions to Caretakers	Special category
Patient personal hygiene inadequate	Follow up after one day of treatment to assess the radiation safety practice followed or reinforce the education	Patient education radiation safety
Staff not surveyed before leaving room	Staff Survey by a two‐person team	Contamination control
Staff not surveyed before leaving room	Place a reminder on the door for each user to be surveyed before leaving the treatment area or put a sign‐in/sign‐out sheet to remind them to be surveyed before they leave	Contamination control
Covers all “personal performance lapse” failures	Meticulous in‐service training and documentation for anyone involved in the RPT treatment process to avoid any personnel performance lapse.	All steps

## 4 DISCUSSION

The FMEA team, consisting of experts from multiple institutions and disciplines, conducted a comprehensive analysis to identify areas where extra efforts are needed to ensure the safe delivery of widely used FDA‐approved 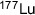
‐based RPTs. The analysis provided valuable insights into the multifaceted nature of potential risks and adverse outcomes. Most institutions participating in the study had high RPN for FMs related to public safety or radiation contamination in the radiation‐related effects segment. These findings highlight the importance of mitigating risks associated with direct patient safety and radiation safety concerns for the public within the radiation medicine landscape of RPTs.

Lutathera and Pluvicto treatments require strict adherence to radiation safety protocols to avoid radiation contamination, which may lead to unintended radiation exposure to the public. The US Nuclear Regulatory Commission (NRC) has established that the public's annual exposure to radiation should not exceed 0.1 rem (1 mSv). Moreover, the dose from external sources in any unrestricted area should not exceed 0.002 rem (0.02 mSv) in one hour.^25^ After treatment administration, a significant amount of 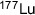 is released through the patient's body fluids and excretory system, which will continue for a few days. Radiation level surveys are a regulatory requirement for patients to be released to the public, along with instructions on following their post‐treatment care. It is important to note that relying solely on checking patient exposure at one meter and ensuring the survey meter reading is less than five mrem/hr before releasing the patient can be risky for public safety. It requires the patient and caregivers to adhere diligently to radiation safety practices after the release from the treatment center. Neglecting these measures can potentially lead to radiation contamination concerns for the public. Inadvertent spillage, inadequate shielding, and incomplete decay in storage before radiation waste disposal can also cause radiation contamination during the RPT handling.^9^, ^19^ Safety measures, like robust patient release protocols, personal protection equipment for healthcare personnel, readily available radiation spill kits, meticulous radiation surveys (both patient and personnel), and patient education, can minimize radiation exposure risks.^8^, ^14^

In the domain of administration‐related effects, a clear trend has emerged from the study. The high RPNs reported by multiple institutions on ‘wasted resources and regulatory non‐compliance’ stress the significance of optimizing resource utilization to enhance cost‐effectiveness and streamline operations. This observation accentuates the importance of operational efficiency and regulatory compliance for RPT programs. It reflects the need for an intricate risk management approach that combines clinical and administrative aspects. Strict adherence to regulatory compliance and treatment delivery protocols following established guidelines is imperative for institutions to protect patient care and institutional reputation during and after Lu‐177 therapy.^8^, ^9^

Among the participating institutions in our study, no reported medical events were associated with 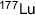
‐based RPTs. In the early years of practice, minor issues such as amino acid shortages and the absence of batch release notes from suppliers were common. However, these problems have diminished over time as experience has increased and the learning curve has been navigated. It is crucial to be aware of the high risk of mixing up Pluvicto and Lutathera doses due to their similar label colors and packaging if they are shipped to the department for use on the same day for different patients, mainly if the dose assay is conducted in haste and without proper attention and training. Administration of these two RPTs for one another on a patient can lead to a severe medical event. We have identified and evaluated this issue as a PFM with step 13 in the survey. Proper training and a clear understanding of the differences between these two radiopharmaceuticals should be sufficient to prevent this potential error. The authors did not encounter dosage errors when assaying the 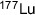 RPTs produced using different methods or laboratories as long as the re‐entrant chamber configuration is according to the manufacturer's instruction. In this study, step 8 of the survey evaluates dosage assay uncertainties using various PFMs. George et al. discuss configurations of dose calibrators for different geometries in currently available 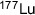
‐based RPTs.^21^

Quantifying the number of failures or near misses experienced by the institutions in delivering RPT is standard practice for retrospective analysis and is not relevant in this prospective study. Solid quality assurance (QA) practices are already in place in all these participating institutions, as outlined in Figures 6, 7, and Table 5, limiting the incidents of failures. Often, near misses or failures are captured on time with a rigid QM program, and the treatments proceed smoothly without incidents or reportable mistakes. Radiation‐related errors rarely occur if the participating institutions implement strict QA practices and training.

An innovative approach is needed to educate patients and caregivers on radiation safety and prevent unwanted radiation exposure to them and the public. Ensuring appropriate transportation arrangements, implementing questionnaires and instructional materials for caretakers regarding radiation safety practices, and conducting follow‐up assessments post‐treatment are recommended. Emergency response teams with specialized training are also beneficial steps toward a robust safety culture.^10^ Furthermore, precise documentation of the treatment procedures, including radiation surveys in the electronic medical record, and meticulous in‐service training for all personnel involved in the RPT treatment process are essential to prevent performance lapses. By incorporating these proactive QM measures, treatment centers can enhance safety and minimize risks associated with 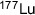 RPT delivery.

The study is based on a prospective analysis of PFMs enlisted by the four participating institutions and a survey conducted at two RO departments, one hybrid NM&RO treatment center, and one NM department where Lu‐177‐based RPT treatments are performed regularly in high volumes. The FMEA and FTA provide a snapshot of the data analysis based on the survey conducted for this study and the experiences of the participating institutions. It is important to note that the Survey may only capture the possible failure modes, which can vary from clinic to clinic. The proposed QM measures may encounter challenges during implementation due to resource differences, treatment volumes, staff training, and institutional policies across various centers and state regulations. Still, it is necessary to conduct ongoing monitoring and updates to adapt to new risks or changes in treatment protocols. A prospective study based on the TG‐100 cannot address all these issues, and individual departments could consider this study as a guideline for continuous improvement in the future to conduct an intra‐departmental prospective analysis and implement or update their respective QM program.

## 5 CONCLUSION

This study applies FMEA and FTA to enhance the QM Program of approved 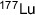 tagged RPTs currently available for clinical use. Compared to other limited numbers of RPT risk analysis studies, we developed a collaborative approach of systemic improvement for 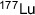
‐based RPTs in a multi‐departmental scenario. We thoroughly examined the FDA‐approved 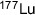
‐based RPTs to identify potential vulnerabilities in administering Pluvicto and Lutathera across healthcare settings. The investigation processed FMEA and calculated RPNs and FTA for a high‐scoring effect. The study proposes improving patient and public safety by introducing or upgrading QM methodologies in RPT treatment delivery. The study's findings sparked in‐depth discussions on refining the 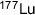
‐based RPT delivery practice across multiple participating institutions and departments. This resulted in a collaborative approach to optimize patient outcomes and enhance safety.

Administering radiopharmaceutical treatments requires a structured approach and the involvement of a multidisciplinary team. Dissemination of knowledge through innovative methods is significant in delivering safe RPT programs. Staff training in clinical judgments and deploying visual tools can enhance the secure environment for treatment delivery. Adopting a holistic strategy to deliver RPTs like Lutathera and Pluvicto can significantly benefit patients, healthcare systems, and management teams. Institutions can optimize patient outcomes and enhance treatment efficacy through this integrative approach.

## CONFLICT OF INTEREST STATEMENT

The authors declare no conflicts of interest.

## CONSENT FOR PUBLICATION

No human subjects were involved in this study. A survey was conducted in participating departments. All 23 survey participants agreed to publish their opinions using the author's scientific analysis. The authors approve the publication of this study.
